# Initial Development and Validation of a Transition Readiness Scale for Adolescents with Inflammatory Bowel Disease

**DOI:** 10.1155/2019/5062105

**Published:** 2019-06-18

**Authors:** Oded Hammerman, Areej Bayatra, Dan Turner, Arie Levine, Raanan Shamir, Amit Assa, Michael Wilschanski, Yaacov G. Bachner, Eran Israeli

**Affiliations:** ^1^Institute of Gastroenterology and Liver Diseases, Hadassah-Hebrew University Medical Center, POB 12000, Jerusalem 91120, Israel; ^2^Institute of Pediatric Gastroenterology, Shaarei Zedek Medical Center, 12 Shmuel Beit St., Jerusalem 9103102, Israel; ^3^Unit of Pediatric Gastroenterology, Wolfson Medical Center, 62 Halochamin St., Holon/Tel Aviv 5822012, Israel; ^4^Sackler Faculty of Medicine, Tel Aviv University, POB 39040, Tel Aviv 69978, Israel; ^5^Institute of Pediatric Gastroenterology, Nutrition, and Liver Diseases, Schneider Children's Medical Center, POB 559, Petach Tikva 4920235, Israel; ^6^Unit of Pediatric Gastroenterology, Hadassah-Hebrew University Medical Center, POB 12000, Jerusalem 91120, Israel; ^7^Department of Public Health, Faculty of Health Sciences, Ben Gurion University of the Negev, POB 653, Beer Sheva 8410501, Israel

## Abstract

**Background and Aims:**

To date, there are no validated measures in IBD to assess the level of preparedness for transition into adult health care. The purpose of this study was to develop and assess the reliability and validity of a “Transition Readiness” (TR) measure for adolescents with IBD, as well as to evaluate the level of TR synchronicity between adolescents themselves, their parents, and their pediatric gastroenterologists.

**Methods:**

A self-assessment tool was created to evaluate TR. Items were reviewed for face validation by IBD experts, and an exploratory factor analysis was performed which yielded 3 distinct domains. The study cohort included adolescents aged 12-21 yrs, their parents, and their physicians in pediatric IBD centers. Correlations between patient/parent/physician TR between each of the domains and the overall TR score to age were assessed.

**Results:**

63 subjects (average age 16.6 yrs/79% Crohn's disease/44% male) participated in this study. There was a significant correlation between the scoring of adolescents and parents on all three domains. The correlation between adolescents and physicians, as well as between parents and physicians, was only consistent for self-efficacy. Self-efficacy significantly correlated with age, while the correlations between perceived knowledge and perception of medical care with age were not significant.

**Conclusion:**

Validation of a novel TR measurement for adolescents with IBD demonstrated a good correlation between patients and parents. Out of the three proposed constructs, perceived self-efficacy is the most salient measure.

## 1. Introduction

As many as 25% of IBD patients are diagnosed by the age of 16 yrs; a life event that will influence patients' across many different developmental realms, such as education, physical growth, and psychological well-being [1, 2]. A successful transfer of adolescents to adult care requires for both pediatric and adult gastroenterologists to understand issues particular to the process of the transition [3].

Important distinctions exist between pediatric and adult care, and each have different areas of focus. Pediatric care is often more focused on healthy growth and development, while adult care concerns itself with other health issues, such as fertility and cancer surveillance [4]. While pediatric care values nurturance and often includes family members, the adult care model values autonomy and respect for a patient's privacy, often with subsequent exclusion of family members. Adult care requires a more active level of participation and self-management by the patient. Decision making, self-advocacy, self-efficacy, communicative skills, medical knowledge, and basic skills, such as the ability to schedule appointments and contact one's provider, are all important aspects of the self-management of IBD, and must exist to some degree if a patient is to transition smoothly to adult health care [4]. It is generally accepted that successful transition programs should promote continuity of care, improve treatment adherence and disease knowledge, encourage self-efficacy and independent disease management, and build confidence in the new adult health-care team, with the overarching goal of improving or maintaining disease control [5]. Patients who are unprepared for this change may face treatment nonadherence, decreased attendance at office visits, and flares in disease activity [6].

However, assessing children with IBD for maturity and readiness to transition to adult care is challenging in light of the lack of validated scales. Many papers have been published on transition in IBD, but only a few have transcended beyond the evidence level of a single center [7]. Studies tend to focus on one key group of stakeholders (e.g., patients) and excluding other important groups with a vested interest in transition (e.g. parents and treating physicians). Without appropriately validated tools to assess readiness for transition, pediatric gastroenterologists may overestimate the readiness of their patients to transfer their care to adult gastroenterologists [8].

The most studied measure of transition readiness (TR) in IBD is the Transition Readiness Assessment Questionnaire (TRAQ), which mainly assesses acquisition of skills for the self-management of the disease (self-efficacy) [9, 10]. This includes tasks such as scheduling appointments, monitoring symptoms, seeking out health care when needed, and taking medication as prescribed. According to a preset threshold of the TRAQ (mastery of 9 out of 20 items), Gray et al. found that only 5.6% of older adolescents/young adults were found to be well prepared for transition. TR was associated with older age but not time since diagnosis, physician global assessment, or confidence ratings [11].

The primary aim of this study was to develop and assess the reliability and validity of a novel “Transition Readiness Measure” (TRM) for adolescents with IBD that would contain additional domains to self-efficacy, thereby delineating some of the factors central to a smooth and healthy transition. In addition, this study aimed at evaluating the level of coherence between adolescents' own evaluation of their preparedness and the estimations of their parents and pediatric gastroenterologists.

## 2. Methods

### 2.1. Participants and Procedure

In order to obtain a heterogeneous sample, patients, their parents, and their physicians were recruited from four different pediatric IBD centers from around Israel (“Hadassah-Hebrew University Medical Center,” Jerusalem, “Shaarei Zedek” Medical Center, Jerusalem, “Wolfson Medical Center,” Holon/Tel Aviv, and “Schneider Children's Medical Center,” Petach Tikva). Inclusion criteria for the study were adolescents being followed in a pediatric clinic between the ages of 12 and 21 yrs diagnosed with IBD (diagnosis >3 months prior to enrollment). This age group was selected for the purpose of observing differences in TR levels over the course of early adolescence on into young adulthood. The study was authorized by the Institutional Review Boards of the participating centers. Adolescents and their parents were enlisted to the study during standard visits to the clinic and were asked to complete the questionnaires on-site. The treating physician also filled out the questionnaire.

## 3. Measures

### 3.1. Transition Readiness Measure (TRM)

The TRM was developed as an instrument to evaluate the readiness of adolescents for transition to adult care. Based on a review of the medical literature, three domains were hypothesized as being important to the transition process and assessing adolescents' ability to become more independent in caring for their illness: perceived knowledge regarding illness (domain 1), perceived self-efficacy regarding illness (domain 2), and perception of medical care (domain 3) [7, 12, 13]. Based on these domains, an initial number of 26 items were generated. These items were then reviewed by 5 pediatricians experienced in IBD with large-volume IBD centers. In order to ascertain face validity, experts were asked to grade each item on a Likert scale of 1-10 based on the items' relevance for a successful transition and the adolescent's ability to become more self-reliant regarding health care. After receiving the experts' scoring and comments, items with an average score lower than 8 were removed from the TRM. Three additional questions were removed as a result of exploratory factor analyses conducted on the measure. The final version included 16 questions rated on a Likert scale ranging from 1 “disagree” to 5 “agree.”

### 3.2. Manitoba IBD Index (MIBDI)

The MIBDI is a validated single-item rating scale which addresses disease activity over the previous 6 months. During enlistment into the study, the participants rated their disease on a 6-point Likert scale, as 1 “constantly active,” 2 “often active,” 3 “sometimes active,” 4 “occasionally active,” 4 “rarely active,” and 6 “inactive” [14].

### 3.3. Demographic Survey

Participants were asked to provide information regarding age, gender, date and place of birth, illness identification (UC, CD, or IBD undefined), and disease duration.

### 3.4. Statistical Analysis

The statistical analysis was generated using SAS software, version 9.4. Continuous variables are presented by mean ± STD. Categorical variables are presented by*N*(%). Exploratory factor analysis was done by the factor analysis procedure with the varimax rotation method. Cronbach's alpha was calculated to assess internal reliability. Pearson's correlation coefficient was used to assess the association between continuous measures. Two-sided *p* values less than .05 were considered statistically significant.

## 4. Results

### 4.1. Patient Characteristics

A total of 63 subjects participated in this study. Corresponding physician-parent questionnaires were obtained for 42 participants. Corresponding physician questionnaires (without the parent questionnaire) were obtained for an additional 7 subjects. For 10 more subjects, the questionnaires were filled out only by a parent, and for 4 subjects, only an adolescent filled out the questionnaire. Demographic and clinical characteristics are presented in Table 1. The median age was 17 yrs with a range of 12-21 yrs. Fifty participants (79%) were diagnosed with CD. Most participants (92%) were diagnosed with IBD for over 2 years at the time of the study. Illness severity over the previous 6 months according to the MIBDI is detailed in Table 1.

### 4.2. Exploratory Factor Analysis and Internal Reliability

The sample was found to be suitable for factor analysis (the Kaiser − Meyer − Olkin measure of sampling adequacy = 0.691). Exploratory factor analysis (EFA) was performed for all groups, limited to 3 factors, but focusing on the adolescent data (Table 2). The EFA results suggested that two items simultaneously loaded for more than one factor, and thus were removed from the analysis. An additional EFA was performed for the remaining 17 items, and an additional item was removed, as it did not distinguish between factors. A final EFA was then performed for the remaining 16 items, and all items were found to definitively belong to one of three factors. In this final EFA, 6 items were found to fit domain 1 (perceived knowledge regarding illness), 5 items were found to fit domain 2 (perceived self-efficacy), and 5 items were found to fit domain 3 (perception of medical care). The internal consistency was good for all three questionnaire domains with Cronbach's *α* ranging from 0.77 to 0.84 across the domains. Cronbach's *α* for the entire questionnaire was 0.84. Factorial loadings as well as internal reliability and Eigen values are shown in Table 2.

### 4.3. Correlation between Factors

Correlations between the three factors of the TRM were relatively low, meaning that the domains tap different aspects of the concept (Table 3).

### 4.4. Association between TR and Adolescents' Age

After making certain that age has a normal distribution (skewness = −0.457, kurtosis = 0.023), correlation between TR domain scores and age were calculated (Table 4). There were no correlations between age and perceived knowledge (*r* = 0.103) or perception of medical care domains (*r* = −0.150). Age had a significant correlation with the perceived self-efficacy domain, and the strength of the association was moderate (*r* = 0.367, *p* < 0.01).

The distribution of mean TR scores by 4 age groups is shown in Table 3. Domains 1 (perceived knowledge) and 3 (perception of medical care) were not associated with age for adolescents, parents, or physicians. Domain 2 (perceived self-efficacy), however, showed a consistent increase in mean TR scores across all age groups and for all populations (adolescents, parents, or physicians), save for a slight decrease in the mean score for adolescents from ages 12-16 (3.15) to ages 16-18 (3.05).

There was a moderate correlation between adolescents and parents on all 3 domains (Table 5). Nonetheless, the correlation between adolescents and physicians as well as between parents and physicians was weak (domain 2).

## 5. Discussion

As young people move towards adulthood, their medical and psychological needs change and it is crucial for them to receive age-appropriate medical care. Preparing young adults for this transition is very important, as they need to develop a sense of independence and maturity [15]. Ideally, transition should occur when young patients possess the necessary skills to function in an adult care setting [16], and the timing of this transfer should be carefully planned together with the patient and their parents [17].

The primary aim of this study was to develop and assess the reliability and validity of a “Transition Readiness Measure” (TRM) for adolescents with IBD. Although the TRM had good internal reliability across all three domains, and consensus regarding the items was achieved by experts in the field of pediatric gastroenterology, some components of the measure remained problematic. Our hypothesis regarding the correlation of TR with age was that if the TRM truly reflected TR, we would see a gradual increase in preparedness across all domains of the questionnaire.

Following an in-depth literature review, three domains were created for the purpose of measuring TR. The 1st domain was created based on the understanding that knowledge regarding IBD is an important factor in transition readiness; however, actual knowledge regarding IBD may not be the most helpful indicator. A study by Huang et al. [8] showed that the percentage of adolescents with IBD who had significant actual knowledge regarding their illness was 0% for adolescents <14 yrs. and 22% for adolescents aged 14-18 yrs. Due to low levels of actual knowledge, this study focused on perceived knowledge, assuming that adolescents who feel they know more about their illness will also feel more in control and be better prepared to take charge of their medical care. It is possible that perceived knowledge did not correlate with age because it is more a function of time since diagnosis than age. However, if this is indeed the case, it would mean that perceived knowledge is not an ideal indicator of TR, as it is associated to other factors. A more plausible explanation is that the participants for all age groups rated their knowledge of IBD as fairly high. The mean perceived knowledge was ≥4 in all age groups except for the 16–17-year-old adolescents. Thus, the mean score for this domain did not show a consistent increase with age. This finding has internal consistency since this age group was also rated the lowest among parents and physicians.

The 2nd domain focused on perceived self-efficacy (SE) regarding health. SE is a person's belief in their capability to produce and execute actions that regulate events in their lives [18]. Self-efficacy skills are likely to include the ability to monitor symptoms and report them to a health-care professional. The patient needs to be able to demonstrate the skills necessary to navigate and communicate with the health-care system, such as making appointments and filling prescriptions. Self-efficacy skills may also include handling financial issues and arranging transportation to and from the hospital. The assumption here is that the more perceived SE adolescents have, the better they will adapt to their illness and take care of themselves in regard to handling their illness. This domain has the highest resemblance to the TRAQ.

The 3rd domain focuses on the relationships patients have with their physicians and their conceptualization of the medical care they have received. The patient-provider relationship has been shown to be a strong predictor of patient adherence to treatment [6]. Our study showed that perception of medical care scores were also found to be inconsistent with age. It is interesting to note that the mean scores for this domain actually ranked highest for ages 12–16 yrs, demonstrating the trust that the patients developed in their pediatric care, due to the supportive model used by pediatric gastroenterologists. The mean scores for this domain remained high (>4) with increasing age.

Thus, the only domain that had external validity as an indication of TR in terms of its' correlation with age was perceived self-efficacy. The hypothesis that SE and self-management skills are an important part of the transition process and lead to transition readiness has been verified previously [1, 19]. Nonetheless, based on these findings can a conclusion be made that transition readiness should only be measured according to SE, and that no additional measure is needed? This hypothesis should be taken with caution, until additional external validity outcomes (in addition to correlation with age) are measured, e.g., disease outcomes and direct behavioral measurements.

As there is no clear age cutoff for transition and different developmental milestones in adolescence may be reached at different times, current practices are often based on the experience of the physicians involved [2]. The current study demonstrated significant correlations between parents and their children's TR scores, while a poor correlation appeared between adolescents' self-scoring and the assessment of their treating pediatric physicians' preparedness. Other studies have similarly found poor correlations between physicians' assessments of their patients and patients' own self-assessments [8]. This may suggest that physicians should not rely simply on their gut feelings regarding TR but should rather make use of standardized assessment tools.

The strengths of this study include inclusion of patients from multiple centers and that the TRM was scored simultaneously by different stakeholders involved in the treatment of adolescents with IBD (patients, parents, and treating physicians).

This study also has a number of limitations. First, our sample size was relatively small and was derived from hospital settings. It is possible that recruitment from hospital settings as opposed to a community health-care setting created some selection bias. This collection method was due to the fact that pediatric patients with IBD in Israel are treated primarily in hospital outpatient centers. In addition, this study did not corroborate the various TR domains with biological or behavioral endpoints. Hence, future studies made with larger and representative sample sizes are needed in order to further investigate which aspects of TR correspond to better disease management and improved medical outcomes.

Our results support the conclusion that in adolescents with IBD, there was a good correlation between self-assessment for TR and that of the parents, but not with the treating physicians. Out of the three domains constructed to assess TR, the perceived SE is the most salient measure. Regardless, and despite the lack of correlation with age, perceived knowledge of IBD is essential for appropriate medical care, as well as patients' thoughts regarding the care they receive.

## Figures and Tables

**Table 1:** Sociodemographic characteristics of the study participants.

	Study group (*N* = 63)
Average age (±S.D.)	16.6 (±2.1)
Country of origin	
Israel	59 (93%)
Other	5 (7%)
Gender	
Male	28 (44%)
Female	35 (56%)
Diagnosis	
UC	12 (19%)
CD	50 (79%)
IBD unclassified	1 (2%)
Time since diagnosis (duration of illness)	
<2 years	5 (8%)
>2 years	58 (92%)
Average age at diagnosis	13.1 (±2.4)
Median age at onset of symptoms	12.4 (±2.9)
IQR at onset of symptoms	4.13
Manitoba IBD index (MIBDI)	
1	3 (5%)
2	15 (24%)
3	7 (11%)
4	8 (12%)
5	14 (23%)
6	16 (25%)

**Table 2:** Factorial weights and coefficients of discrimination for adolescents.

		Factor 1. Knowledge	Factor 3. Medical care	Factor 2. Self-efficacy
Q1	I know the important things about my illness	*0.719*	0.081	0.018
Q3	I recognize when my symptoms indicate that I need to go and see my doctor	*0.825*	0.218	-0.016
Q4	I am interested in and read information about my illness	0.233	*0.601*	0.193
Q8	I understand what symptoms are part of my illness	*0.725*	0.163	0.188
Q18	I know when I should turn to the medical staff for help	*0.784*	0.038	0.211
Q2	I make sure to take my medications on my own	0.119	*0.672*	0.203
Q5	I set my doctors' appointments on my own	0.108	0.186	*0.598*
Q6	I'm comfortable going to the gastroenterology clinic by myself	0.205	0.279	*0.785*
Q9	I do not need my parents help to take care of my illness	0.033	-0.187	*0.756*
Q13	I feel confident going to the doctor on my own	0.243	0.134	*0.812*
Q15	I know how to explain my symptoms to my doctor	*0.581*	0.056	0.249
Q20	I know how to go to the doctor when I need help	*0.601*	0.261	0.172
Q25	I can take care of my health on my own	0.108	-0.063	*0.564*
Q10	I believe that my doctors are helping to keep me healthy	0.110	*0.726*	0.052
Q16	I understand that I should follow the doctor's instructions about how to take my medication	0.063	*0.788*	-0.054
Q24	I listen to and apply what the doctors tell me	0.153	*0.749*	-0.118
	Eigen values	*4.892*	*2.301*	*1.712*
	Cumulative % of explained variance	*20.4*	*38.2*	*55.6*
	Cronbach's *α* per factor^∗^	*0.840*	*0.785*	*0.774*
	Cronbach's *α* for all 16 items^∗^	*0.840*		

^∗^Standardized and corrected for the number of items within the scale.

**Table 3:** Correlation between domains of the TRM.

	Knowledge of IBD (1)	Medical care (3)	Self-efficacy (2)
Knowledge of IBD (1)	1	0.453^∗∗^	0.405^∗∗^
Medical care (3)	0.453^∗∗^	1	0.266^∗^
Self-efficacy (2)	0.405^∗∗^	0.266^∗^	1

^∗^ *p* < 0.05 and ^∗∗^*p* < 0.01.

**Table 4:** Mean TR scores by age.

Group	Age	*N*	Domain		
			Perceived knowledge of IBD (1)	Perceived self-efficacy (2)	Perception of medical care (3)
Physicians	12-<16	11	4.0 ± 0.9	2.7 ± 0.7	4.6 ± 0.6
	16-<18	18	3.7 ± 0.9	2.9 ± 0.8	4.0 ± 0.9
	18-≤19	10	4.6 ± 0.4	4.0 ± 0.5	4.9 ± 0.2
	20-23	1	4.4	4.8	4.3

Adults	12-<16	14	3.9 ± 0.5	2.8 ± 0.8	4.5 ± 0.8
	16-<18	20	3.9 ± 0.8	3.0 ± 0.9	4.0 ± 1.0
	18-<19	31	4.3 ± 0.7	3.2 ± 0.7	4.3 ± 0.6
	>19-23	16	4.2 ± 0.6	3.6 ± 0.8	4.3 ± 0.7

Adolescents	12-<16	12	4.2 ± 0.4	3.2 ± 0.6	4.6 ± 0.3
	16-<18	21	3.8 ± 1.0	3.1 ± 0.9	4.3 ± 0.6
	18-<19	30	4.2 ± 0.8	3.5 ± 0.7	4.5 ± 0.5
	>19-23	15	4.1 ± 0.5	3.6 ± 0.6	4.2 ± 0.8

^∧^PTT score ranges from 1 to 5.

**Table 5:** Correlation of TR domains between adolescents, parents, and physicians.

	Adolescent to parent	Adolescent to physician	Parent to physician
Knowledge of IBD (1)	0.57961^∗∗^	0.22281	0.24914
Medical self-efficacy (2)	0.65726^∗∗^	0.35496^∗^	0.37464^∗^
Perception of medical care (3)	0.60489^∗∗^	0.27896	0.31951^∗^

^∗∗^ *p* < 0.001 and ^∗^*p* < 0.05.

## Data Availability

The data used to support the findings of this study are included within the article.
